# Multistable autonomous motion of fruit on a smooth hotplate

**DOI:** 10.1038/s41598-021-03859-8

**Published:** 2022-01-07

**Authors:** Promode R. Bandyopadhyay

**Affiliations:** Zwim Robotics, 153 Chases Ln, Middletown, RI 02842 USA

**Keywords:** Biophysics, Engineering

## Abstract

Origin of scale coupling may be clarified by the understanding of multistability, or shifts between stable points via unstable equilibrium points due to a stimulus. When placed on a glasstop hotplate, cobs of corn underwent multistable autonomous oscillation, with unsteady viscous lubrication below and transitional plumes above, where the buoyancy to inertia force ratio is close to ≥ 1.0. Subsequently, viscous wall-frictional multistability occurred in six more types of smooth fruit with nominal symmetry. Autonomous motion observed are: cobs roll, pitch and yaw; but green chillies, blueberries, tropical berries, red grapes, oblong grapes and grape tomatoes roll and yaw. The cross products of the orthogonal angular momentum produce the observed motion. The prevalence of roll and yaw motion are the most common. Lubricant film thickness *h* $\propto$ *U*/(*TF*), for cob mass *F*, tangential velocity *U* and temperature *T*. In heavier cobs, the film thins, breaking frequently, changing stability. Lighter cobs have high *h*, favoring positive feedback and more spinning: more *T* rises, more viscosity of water drops, increasing *U* and *h* more, until cooling onsets. Infrequent popping of the tender corn kernel has the same mean sound pressure level as in hard popcorn. The plume vortex jets lock-in to the autonomous rolling cob oscillation. Away from any solid surface, the hot-cold side boundary produces plumes slanted at ± 45°. Surface fencing (13–26 μm high) appears to control motion drift.

## Introduction

Following the discovery of multi-periodicity in muscles, lasers and seismology, interest has grown in their theoretical underpinnings and control^1–3^. The understanding of multistability may clarify the origin of large and small scale coupling^4^. The coupling of scales has been reported in turbulent boundary-layers (TBL, Small from Large)^5^ and other shear flows, although the origin is not clear^5–9^. The fluttering instabilities in a two-link mechanical mechanism in the horizontal plane, when subjected to kinetic friction, have been demonstrated^10^. Lorentz–Malkus water wheels produce multistable oscillation^11,12^. During maneuvering, swimming animals change their flapping frequencies.

**Figure 1:**
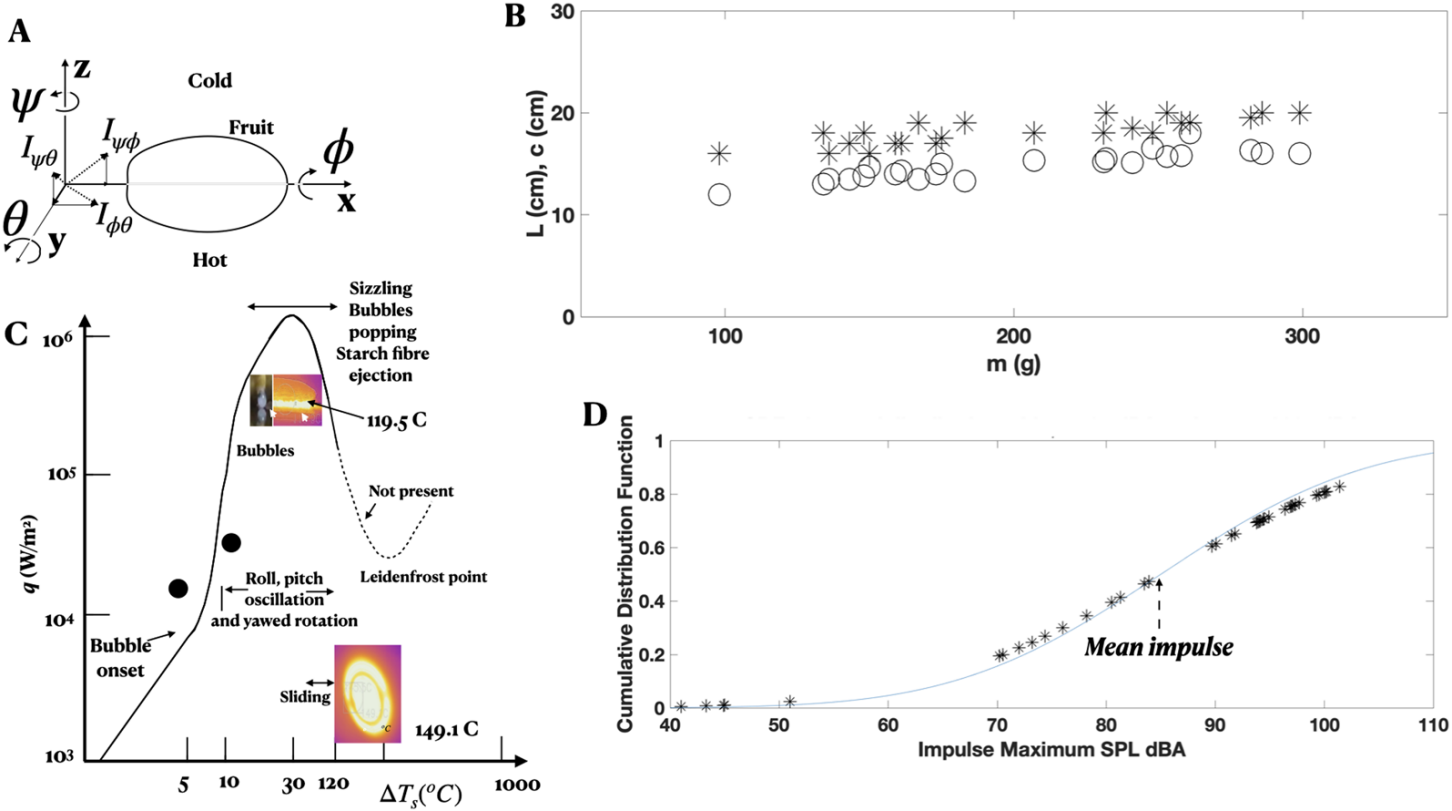
Background. (**A**) Coordinate system of fruit on a hotplate where $I_{\psi \theta }$, $I_{\psi \phi }$ and $I_{\phi \theta }$ are total angular momentum resulting from the orthogonal coupling (cross-product) of components in one Cartesian plane. (**B**) Length (L*) and maximum circumference (*c* O) of the cobs, which are locally axisymmetric. Motion repeatability: all 30 cobs produced autonomous motion upon teasing or re-positioning, moistening and fresh harvesting may have helped; green chillies needed a lower wall temperature; in other types of fruit, a quick surface scalding is avoided in order to generate motion and 50–60% did not produce motion; aspect ratio *a*: an 80 g, 8 cm diameter, $a = 1$ tomato vibrates at high *f* and low *A* for 5 s; but, an 108 g, *a* = 9 cm/6 cm tomato rolled for 18, 19 s at wall temperature of 149.1 °C (Fig. 5); trajectories do not repeat due to initial condition dependence (Fig. 2F, VIDEO 0 COMPOUND PENDULUM). (**C**) Heat modeling (filled symbols) compared, solid line: graph of boiling water due to Incropera^31^. (**D**) Impulsive maximum sound pressure levels of kernel pops: solid line is error function.

By happenstance, it was found that when a cob of corn is placed on a glasstop hotplate, the cob oscillates autonomously about three axes (Fig. 1) with varying amplitudes *A* and frequencies *f*, shifting randomly with time *t*. It begs the questions: How does the cob multistability affect the stability of plumes above and kernel friction below? Are such autonomous oscillations present in green chillies, berries, grapes and tomatoes also? And which motion axes preferably couple (defined as the cross product of orthogonal angular momentum)? See Videos 1–3, 3A,B, 4–8 (Google Drive Link). Cob-plume lock-in (state variables return to initial condition) and the forward motion of pitching cobs have relevance to autonomous swimming of animals and stratification instability to weather and mantle convection. Recent work on popcorn popping appear elsewhere^13,14^.

Hénon maps describe multistable oscillators. Theoretically, attractors are controlled in two ways. In feedback control, continuous inputs bring errors to zero. Alternatively, introducing a predetermined small oscillation cancels an attractor by resonant modulation^2,3^. In yet another alternative, here, the effects of naturally occurring impulsive perturbations on the cob angular momentum are shown, directly relating the cause and effect. The experimental detail is given in the “Methods” and in the Supplementary Information (SI).

## Results

**Figure 2:**
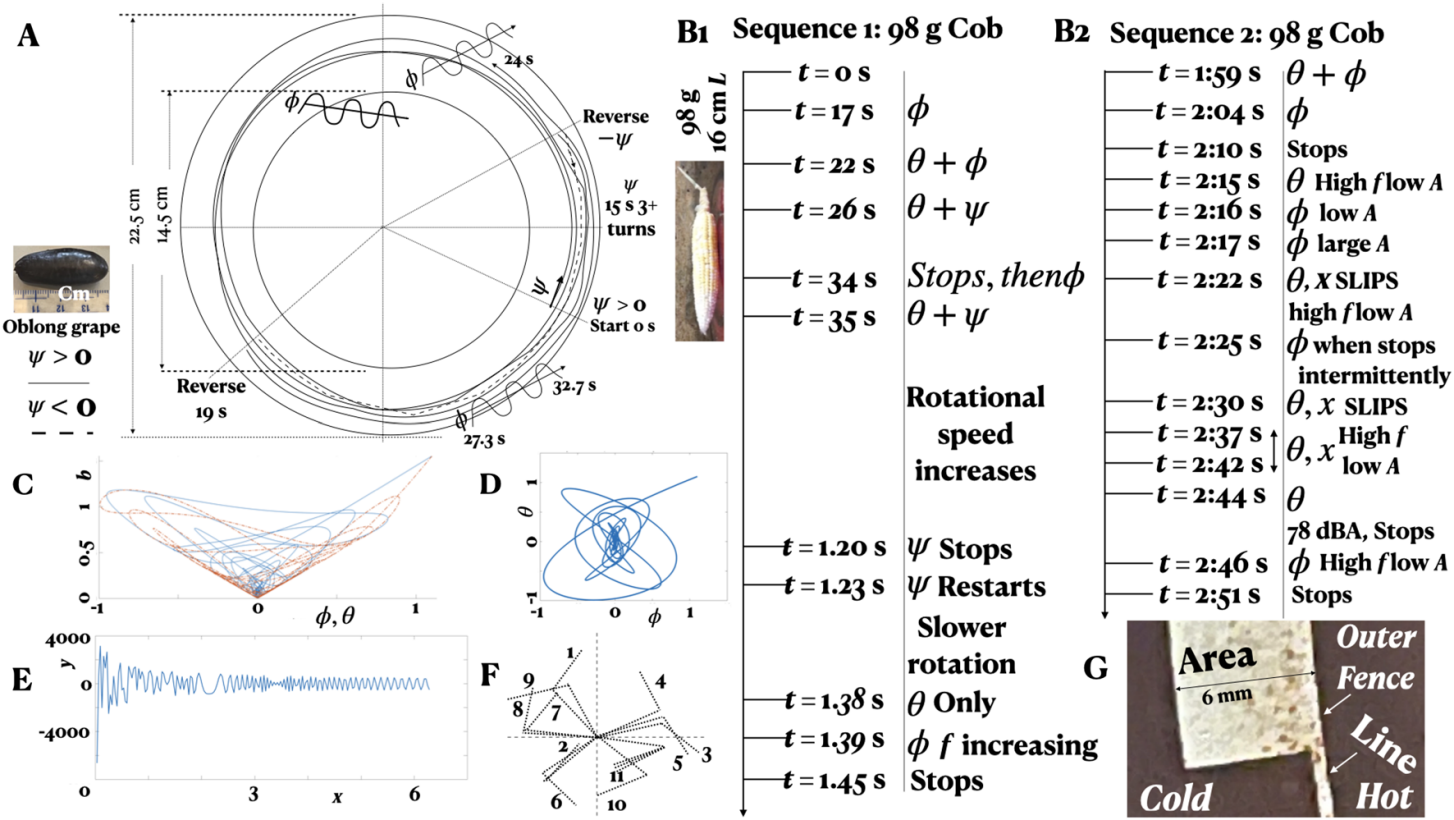
Multistability. (**A**) Oblong grape. (**B1**, **B2**) small cob. (**C**, **D**) Spring, mass and damper model of fruit $\phi$ and $\theta$ oscillations while dampening down. (**C**) Phase plot of net oscillation *b*. (**D**) Flipping in $\phi$ versus $\theta$ as in Lorentz oscillators. (**E**) Phase (*x*) trace of asymmetric sine function of amplitude *y*, $x \rightarrow 2\pi$ in arbitrary scale. Compared to Fig. 4C, in Fig. 2A, $R_{\psi drift}$ $\rightarrow$ 0. (**F**) Asymmetry in experimental 2-link compound pendulum, every 10th frame, 60 frames/s, from VIDEO 0. G: Fence; using a 0.0005” resolution dial gauge validated to 100 μm thick 80 g/m$^2$ white paper, height of frayed painted ring markings ($\epsilon$, also felt by touch), clockwise from North at 45 increment: inner fence 13, 13, 4, 4, 13, 26, 32, 7; outer fence 26, 26, 13, 4, 13, 26, 26, 13 μm, $\epsilon _{area} > \epsilon _{line}$; human tactile sensation threshold 3.3$^{\pm 0.10} \,$μm^32^.

**Figure 3:**
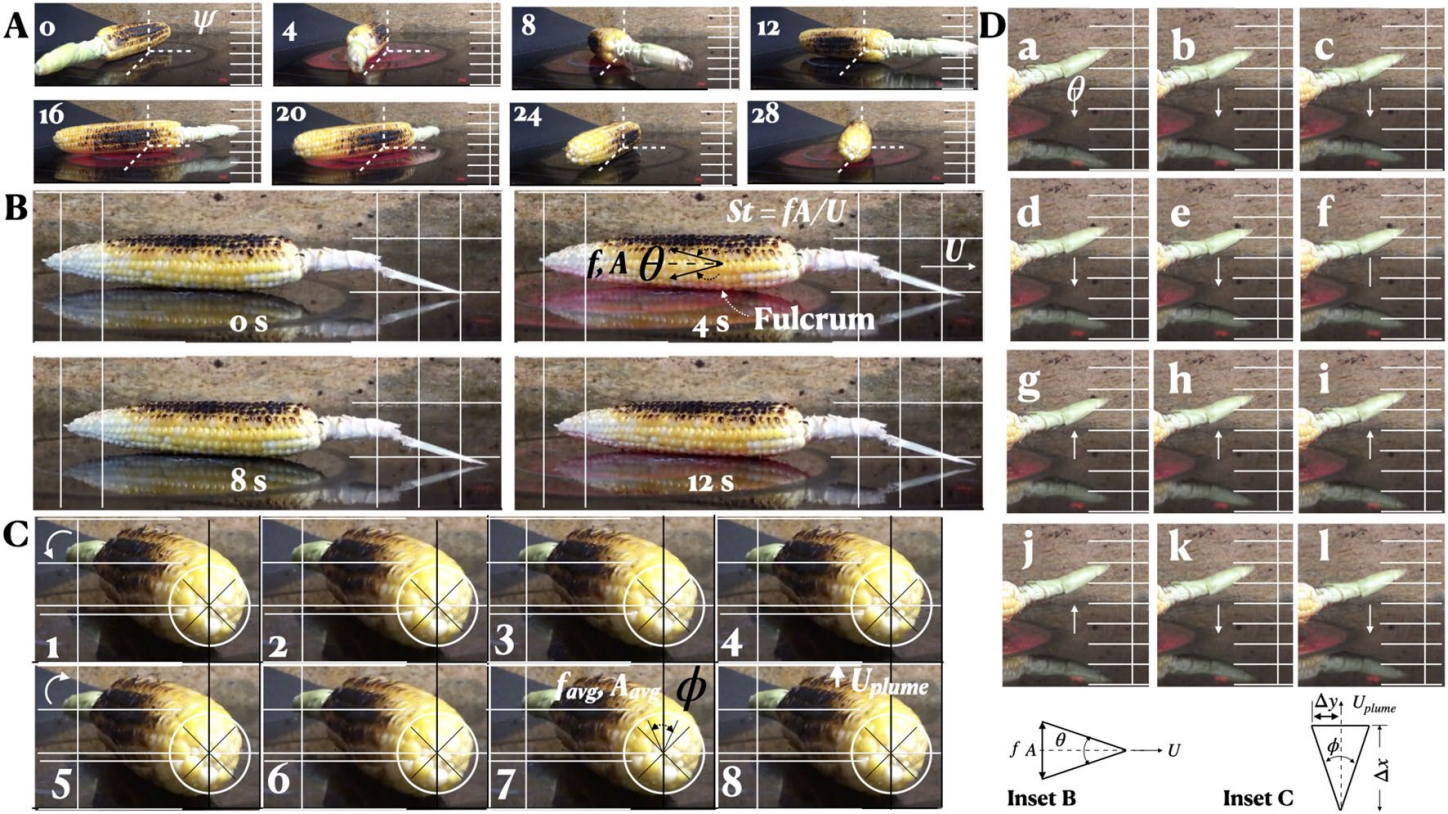
Cob autonomous motion. (**A**) Yawed rotation ($\psi$). (**B**) Longitudinal sliding (*x*) during pitching oscillation. (**C**) Asymmetric rolling ($\phi$). (**D**) Asymmetric pitching ($\theta$). Frames are at equal time intervals. Grid lines are fixed to background. (**B**) Cob $St_{cob}=fA/U$. (**C**) Plume $St_{plume}= 2 \tan {(\Delta y/\Delta x)}$, for small $\phi /2$ (5–10° , Fig. 6c), avg = average value. For conserved *fA* and friction $\rightarrow 0$, $U = U_{plume}$.

**Figure 4:**
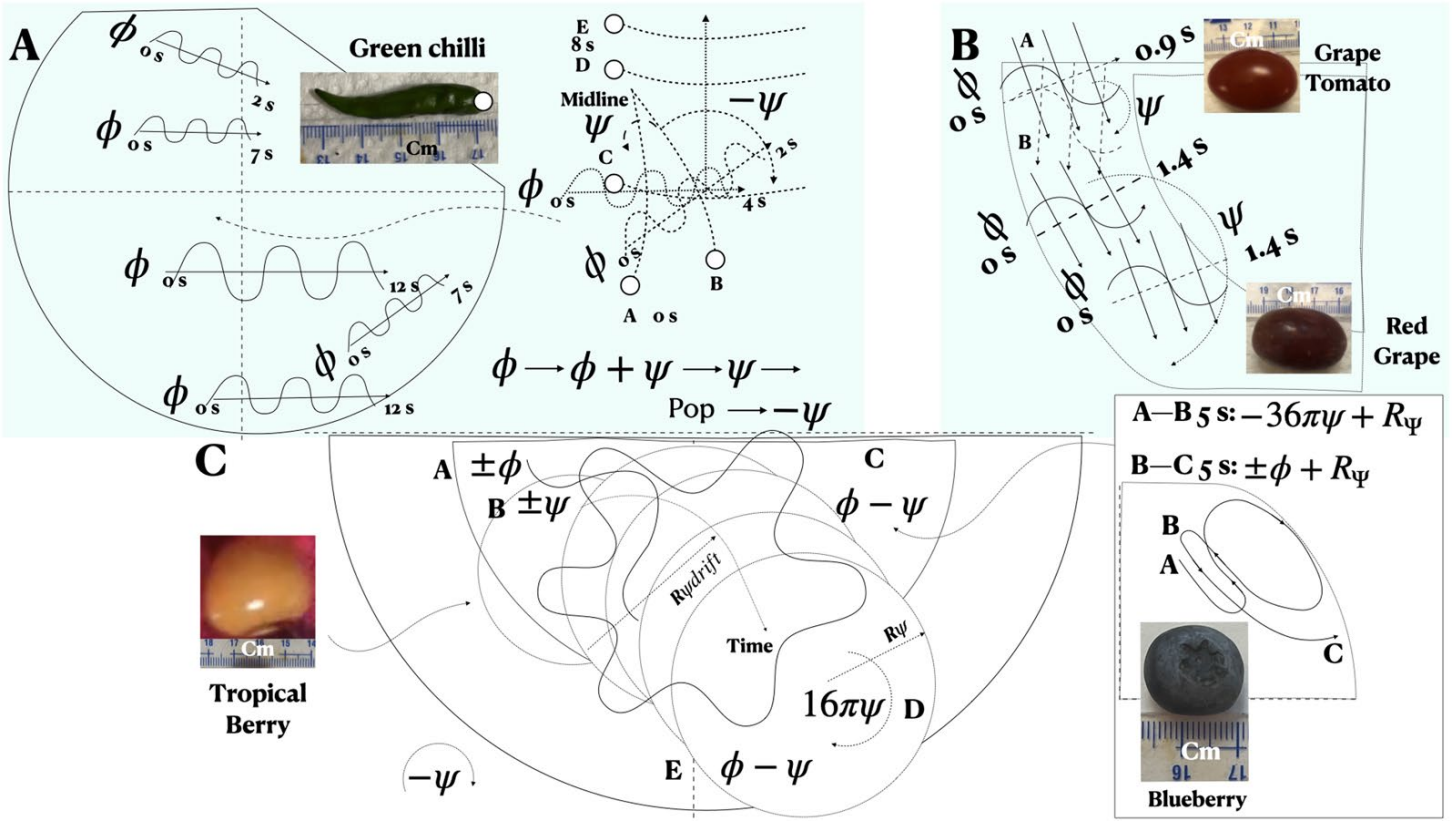
Multistable autonomous trajectories. (**A**) Green chilli. (**B**) Grape tomato, Red grape. (**C**) Tropical berry and blueberry; (**C**) box inset on right: Blueberry; number of full rotations counted by overlaying a fixed reference crosshair on the video frame and reducing the footage speed by x8. Time increases from smaller font A to B to C and so on in each subfigure.

**Figure 5:**
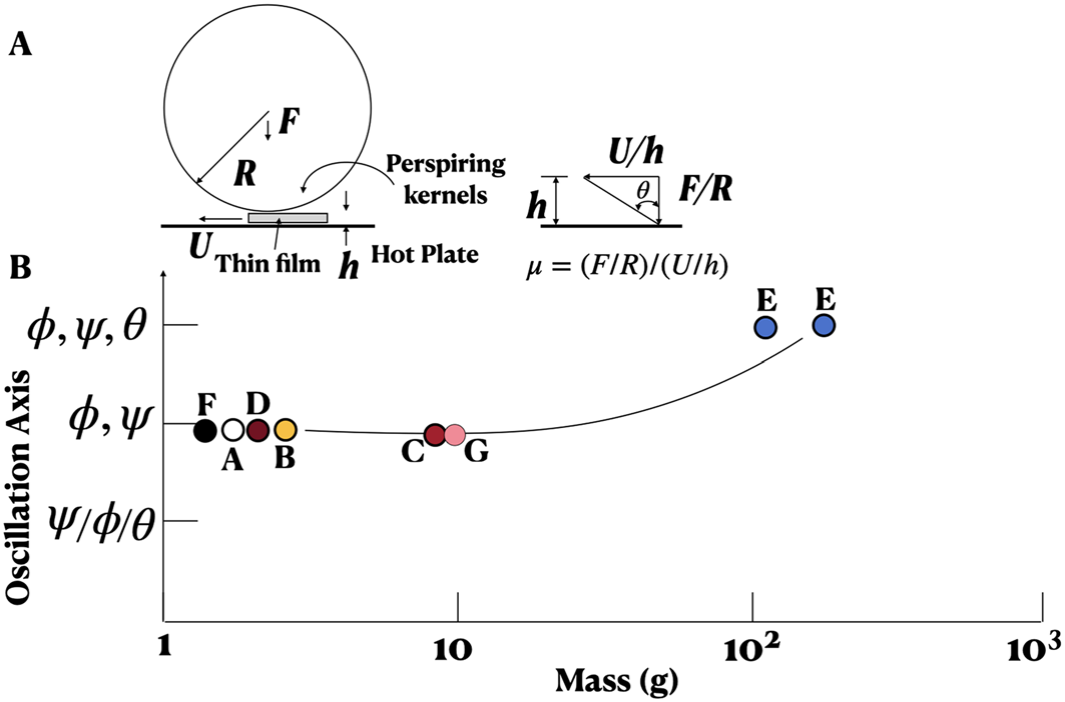
Multistability mechanism and summary. (**A**) Schema model of lubricated wall-frictional mechanism. (**B**) Summary of multistable autonomous motions and oscillations of small fruit. Circles in (**B**) are smaller font (**A**) blueberry tender (aspect ratio = 1.0, round); (**B**) tropical berry firm (1.0, round); (**C**) grape tomato (1.5, tapered); (**D**) oblong grape firm (2.42, tapered); (**E**) corn on cob firm (3.5 to 4.5, tapered and cambered); (**F**) green chilli firm (6 to 10, tapered and cambered); (**G**) red grape firm (1.5, tapered). Nominal symmetry, local in cobs, a surface tension effect, allows independence of shape and size.

**Figure 6:**
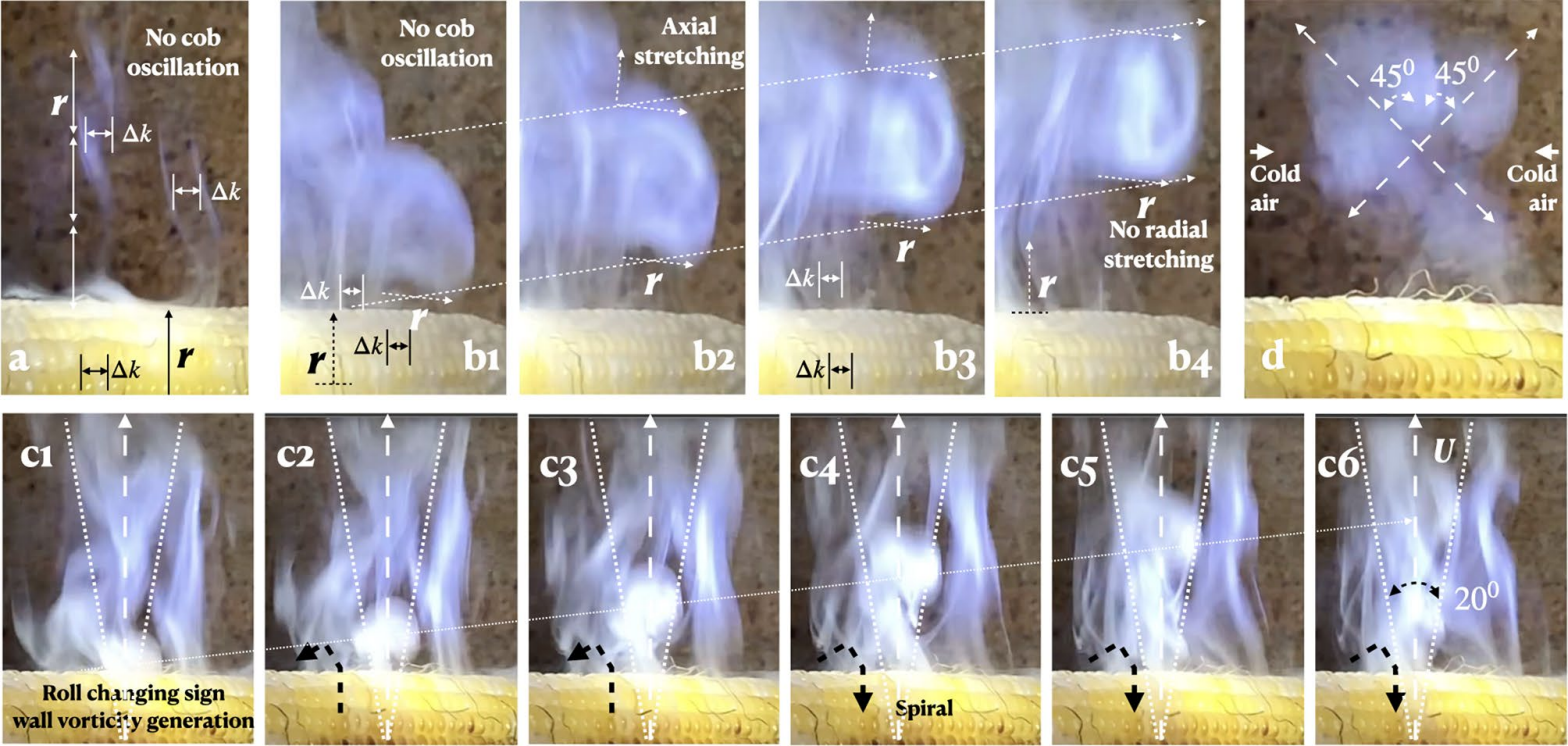
Plume instability, vortex formation and scaling. (**a**) The early stage of instability; laminar twisted vortex tube plumes in cobs not in motion scaling with *r*, local cob radius and $\Delta k$ = width of $two$ kernels. (**b**) Vortex formation in rising plumes. (**c**) Rising plume in $\phi$-oscillation of cob at *f* and *A*; the plume half angle is 10° (compare to Fig. 3, Inset C). (**d**) Vortex stretching along the direction of the principal strain in the main plume in the middle of the cob.

**Figure 7:**
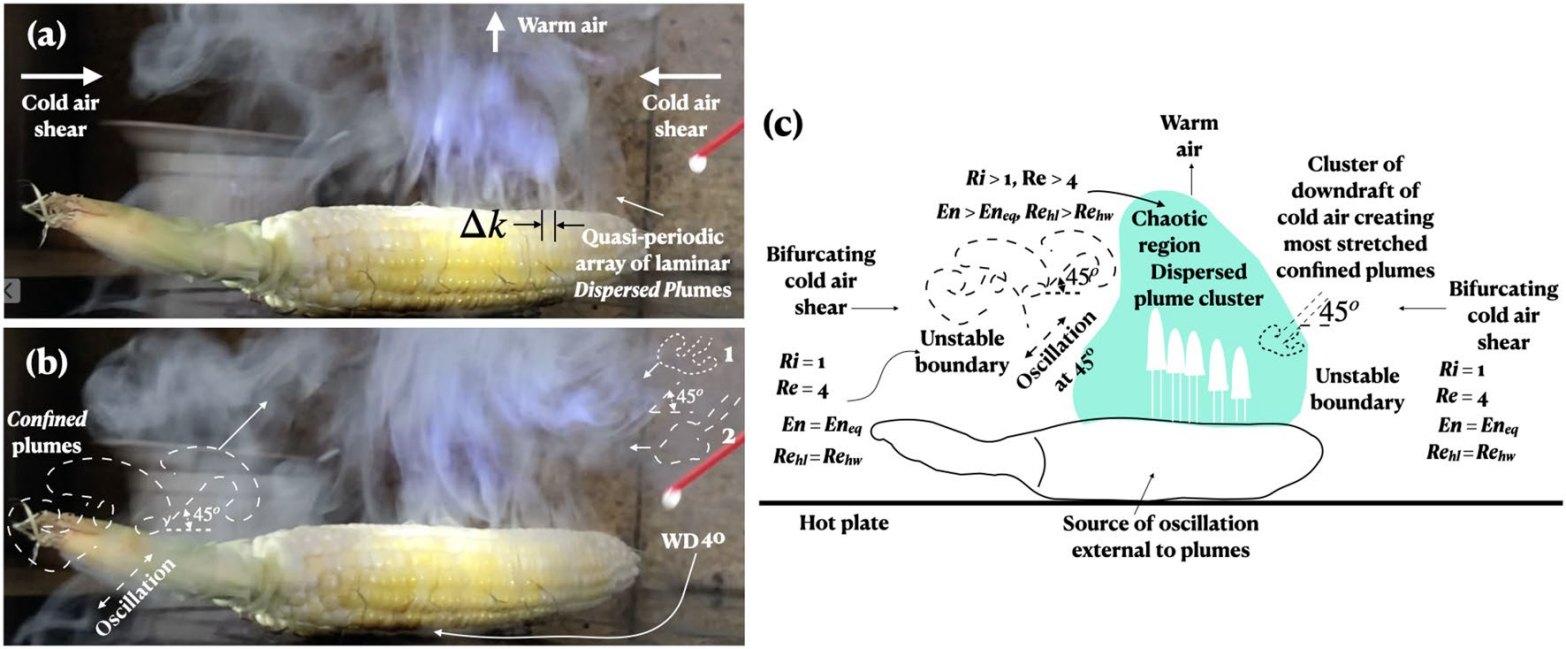
Co-existence of multiple plume instabilities. (**a**) Plume array from kernels; *Ri* > 1.0 (middle), *Ri* = 1.0 (sides). (**b**) Unstable plumes in hot-cold side boundaries; numbers 1 and 2 on right show plume multistability; also shows advancing disorganization^33^ in the middle plume cluster; however, only multistability is seen in the sides, triggered by a small increase in *Ri* from 1.0 and *Re* from 3.0. (**c**) Summary schema demonstrating the different stages of instability advance to chaos in varying physical domains, all co-existing. *En* is plume vortex entrainment and $En_{eq}$ is its equilibrium value at the critical point *Ri* = 1; $Re_{hw}$ and $Re_{hl}$ are Reynolds numbers based on the width and length of the plume head vortex (Fig. 6b)^20^.

Figure 1 gives the background of the experiment. The viscous wall-frictional multistability results appear in Figs. 2, 3, 4 and 5 and the plume instability results appear in Figs. 6 and 7.

### Multistability

Figure 1A shows the Cartesian coordinate system: rolling $\phi$ is rotation about the longitudinal axis *x*, yaw $\psi$ is about the vertical axis *z* and pitching $\theta$, orthogonal to $\phi$ and $\psi$, is about the transverse axis. For symmetric oscillation, $\phi = \phi _o (1+sin (\omega t))$, $\omega = 2\pi f$, where *f* (Hz) is frequency, $\omega$ is in rad/s and subscript o is for amplitude. For asymmetric oscillation, a bias and a phase difference between the orthogonal oscillations may be present. Videos 1 and 2 show symmetric $\phi$ and $\theta$ cob oscillation.

In the cob, ($\psi$, $\theta$), ($\psi$, $\phi$), and ($\phi$, $\theta$) combinations are present (Figs. 2B1,B2). In the tropical berry, and in the remaining five types of fruit, ($\phi$, $\psi$) combinations are present (Fig. 4C). In Fig. 1, say $I_\omega$ is the eigen vector of the angular momentum, which is conserved. Then, in the *xy* plane, if the component $I_\phi$ starts decreasing, the component $I_\theta$ will increase. In other words, the $\phi$ oscillations will gradually shift to $\theta$ oscillation. This shift is what we see in the 98 g cob Video 7. After a 89.7 dBA pop, $\theta$ shifts to $\phi$ in the 200 g cob (Video 7). Similarly, in the *xz* plane, consider the angular momentum eigen vectors $I_\psi$ and $I_\phi$. The tropical berry has $\phi$ and $\psi$ rotations over phase $16\pi$, translating over the *xy* plane very slightly between each full $\psi$ rotation. This rolling yawed rotation signifies that $I_\psi$ and $I_\phi$ remain nearly constant without any transfer of momentum (except that $R_{drift}$ > 0, Fig. 4C). In our Stuart-Landau modeling of low Reynolds number turbulent boundary layers, the assumption of a coupling of the longitudinal vorticity $\omega _x$, with the cross-stream component $\omega _z$, in the wall-parallel plane where turbulence production takes place, has led to a remarkable agreement with spatiotemporal visualizations^5^.

Figure 3C and D show asymmetric $\phi$ and $\theta$ oscillation in the cob: in 3C, compare frames 3 and 7 where the displacements of the stem tip from the nearest vertical line are different in the two; and in 3D, where the upward and downward motion phase changes at frame f and between j and k, the stem tip is higher in k than in e. The 10 mm axial crawl at a snail’s pace with one stop is asymmetric pitching with film break (Fig. 3B, Video 5). Figure 2C,D shows the flipping motion of a dampening spring-mass-damper system as in Lorentz oscillators. Figure 2E shows an asymmetric oscillation given by $y=200*2\pi /\sqrt{(}x))sin(200*2\pi /\sqrt{(}x))$ for phase *x*, see experimental example Fig. 2F. They capture many features of the multistable motion: both abrupt, gradual and repeated changes in *f* and *A*, and sudden stops and starts. *The cob’s forward motion with pitching originates in a bifurcation of lubrication instability, also similar to swimming animal propulsion*^15,16^.

The representative histories of the trajectories of the autonomous motions of these seven types of fruit are shown in Figs. 2, 3 and 4. The oblong grape (Fig. 2A) has the most stable $\phi$ and $\psi$ motion (the trajectory radius variation <5 mm). This fruit rolls in the annular passage between the inner and outer ring paint markings (height 13–26 μm, Fig. 2G) in spite of some debris accumulation. When the counterclockwise path is obstructed by another tender grape, its rotational direction switches to clockwise and the grape resumes another stable autonomous rolling yawed rotation.

Figure 2B shows a diverse portfolio of motion of the lowest mass cob (98 g): the simultaneous presence of $\phi$ and $\theta$ with $\psi$, stops and starts, faster and slower $\psi$ rotations, $\theta$ and $\phi$ monostability, wherein *f* and *A* change inversely, high *f* and low *A* of $\theta$ producing two bursts of < 0.5 cm long *x* motion and popping, which stops any motion. The $\psi$ rotation of the 98 g cob (Fig. 3A) is × 10 faster than in the 200 g cob (Video 5).

*Oscillation.* The autonomous $\phi$ oscillation, with inversely shifting *f* and *A*, is the most common motion among the six smaller and lighter types of fruit (Fig. 4). While most green chillies only have $\phi$ oscillations, the inset of Fig. 4A shows both $\phi$ and $\psi$ motions in one example. In the $\phi$ + $\psi$ motion of red grape and grape tomato, the $\phi$ motion dominates (Fig. 4B). In the tropical berry (Fig. 4C), the otherwise stable motion of $\phi$ + $\psi$ has a secondary overlaid $\psi$ motion whose radius $R_{\psi drift}$ > $R_\psi$ arising from the non-axisymmetry of the berry. However, $R_{\psi drift}$ = 0 in the oblong grape possibly because it is guided by the small (nonuniform, Fig. 2G) paint bumps of the round hotplate’s inner and outer markings. This lateral drift suppression by the surface bumps is similar to the riblet effects on shark skin that reduce TBL streak wandering^5^. In the well lubricated blueberry, $\psi$ and $\pm \phi$ motions are present ($R_{\psi } (t)$> 0, < 0; Fig. 4C box inset on right).

(1) Overall, with the ubiquitous organized autonomous oscillation, the coupling (defined as cross product) of orthogonal *I* components, the conservation of *I* and the shifts in the modes of oscillations are the key features of the fruit motions. (2) The coupling of the fruit oscillations with the plume (defined as lock-in here; below) is another. (3) Another notable result is the following. The two examples where the autonomous $\phi$ or $\theta$ oscillations produce *U*(*x*) or $U_{plume}(z)$ are analogous to autonomous fish swimming (Fig. 3B,C, Inset B, Inset C). Since an *U* along *x*, or $U_{plume}$ along *z* is generated by the *autonomous* $\theta$ or $\phi$ oscillation, *U* based *St* $\approx$ $U_{plume}$ based *St*^17^.

*Popping*. Figure 1D shows the measurements of the impulsive SPL (dBA) of pops which shows that the mean $\mu$ = 85 dBA and the standard deviation $\sigma$ = 14.87 dBA. The estimated sensor margin is ±1.5 dBA. The estimated kernel heat content is in agreement with the boiling heat flux *q* (W/m$^2$, Fig. 1C) and the heat transfer based corn grilling efficiency is then 0.16 (“Methods”). The mean value of $\mu$ is exactly the same as in regular popping popcorn^18^ hinting that the mechanism is the same irrespective of the hardness of the shell—a surprise. While most popcorn pop, however, few of the kernels on the cob do.

As in popcorn, the popping mechanism is based on temperature, not pressure. The weak sensitivity to pressure is also shown by the Clausius–Clapeyron equation^14^. Figure 1D suggests there is a distribution of popping bubble diameter ($d_b$) correlating with the graph of boiling water ($q - \Delta T_o$), the heat flux (*q*, W/m$^2$) versus excess temperature above 100 °C –$d_b \propto \exp ^{-\Delta T_s^2}$ (Fig. 1C). Surface tension initially resists the rising internal pressure holding $d_b$, allowing a build up, then failing suddenly. The 104.1 dBA of the loudest pop measured comes from the smallest $d_b$, a submicron value. More pops for > 85 dBA than for < 85 dBA correlate with an intriguing paucity of data over 85-90 dBA. The bubble may not form immediately post $q_{max}$ where the sign changes to $dq/d\Delta T_s < 0$; there is a transition from nucleate to transition boiling with higher kinetic energy of bubbles past $q_{max}$. The known characteristic sound of intense sizzling post-$q_{max}$ is indeed heard.

For conserved cob angular momentum $I_\omega$, moment of momentum $I_\omega = r \times p$, where *r* is the distance vector of the point from the center of rotation and *p* is the momentum *mv*. Stability shifts suddenly when popping sound pressure $\ge$ mean $\mu$ of 85 dBA (Video 8). When $I_\omega /T \ge 1.0$ for ejected fibre torque *T*, bifurcation shifts the mode.

For cob mass force *F*, tangential velocity *U*, viscosity $\mu$, temperature *T*, from Newton’s friction law, lubricant thickness $h \propto \mu U/F \propto U/(TF)$ (Fig. 5A). The thin water film breaks frequently in heavier cobs changing stability. Lighter cobs have high *h* favoring stability, a positive feedback loop and fast rotation: *T* rising, friction drops, increasing *U* and *h*, converse trend following (Video 5). For pitching: $I_\theta = I_\phi \times I_\psi$, rolling: $I_\phi = I_\theta \times I_\psi$, and $\psi$-rotation, $I_\psi = I_\phi \times I_\theta$ lifts or sinks the fruit.

*Bouncing.* The bounce of a projectile of hot corn starch fibre (Fig. SI-1, Video 6), ejected from a kernel, shows the elasticity of the hot kernels. Elasticity converts kinetic energy into elastic potential energy. Initially, the fibre bounces off the glasstop. Then, it is propelled over the glass surface until it stops becoming a ball. The force of restitution restores the original shape of the fibre and is measured by the coefficient $e=\sqrt{(h_2/h_1)}$, where $h_1$, $h_2$ are the heights of bounce before and after the string hits the glasstop the first time. Similar to a tennis ball’s bounce, *e* = 0.85^19^. All seven types of hot fruit bounce.

The autonomous motions observed are: cobs ($\phi$: Video 1), ($\theta$: Video 2) and ($\psi$: Video 5); green chillies ($\phi , \psi$: Video 3B), blueberries ($\phi , \psi$: Video 4), tropical berries ($\phi , \psi$: Video 4), red and oblong grapes ($\phi , \psi$: Video 3A), and grape tomatoes ($\phi$, $\psi$: Video 3). The prevalence of $\phi$ and $\psi$ is most common. The multistability summary is given in Fig. 5B. There is a correlation with mass. The fruit oscillates when the ratio of inertia to gravity forces is 1.0, nonlinearity ensuing when >1.0.

### Plumes

The plume is visualized with WD-40 spray, comprised of a long-chain hygroscopic hydrocarbon (Figs. 6 and 7). The column diameter exactly matches $\Delta k$, the spacing of two kernels. Vertically, the column undulation in (6a) scales with *r*, the cob radius. Unlike in isothermal plumes due to Rogers and Morris^20^, in (6b), the plume width does not alter, but is stretched axially. The column undulation has doubled. The plume vorticity is generated impulsively on the surface (6c1), the jet angle being ≤ 20°. With cold air side boundaries closing in (6d), the plume slants at 45° to the vertical, maximizing vortex stretching^21–24^.

For small reverse Kármán half plume angles 5°–10° (Fig. 6), 2tan(10°) = $St_{Plume}$ = 0.36—in the range of swimming and flying animals, 0.20–0.40^17,25–27^. Say, $St_{Cob}$ is the cob Strouhal number. For autonomous $\phi$-oscillation, cob and plume are locked-in giving $St_{Plume}$ = $St_{Cob}$ (“Discussion”, Fig. 3). For cob frequency $f$ and amplitude *A* of $\phi$-oscillation, ${fA}$ = constant since $U_{plume} =$ constant. During stable oscillation, net angular momentum is conserved, and *f* $\propto$ 1/*A* (Videos).

Define Prandtl number as the ratio of momentum diffusivity ($\nu$ m$^2$/s) to thermal diffusivity (*K* m$^2$/s), $Pr=\nu / K$. In the kernel water, ${Pr}$ = 7.0 and $\nu$ will dominate over $K$, and convection may be present. With the gaseous plume in Figs. 6 and 7, ${Pr}$ = 0.71 and both momentum and heat will dissipate at nearly the same rate allowing visualization.

The analogous mechanisms of the diffusion of heat, vorticity and momentum above the surface give insight into the lock-in of plume vorticity and autonomous cob motion due to heat. See “Discussion” below and Fig. 6c. Due to surface heating and the cob’s motion, vorticity is produced on the surface. The length $L$ and time $t$ scales of the diffusion of vorticity and heat on the cob surface are estimated by considering the heat and vorticity equations. Say *T* is the temperature and $\omega$ is the vorticity. The vorticity equation is $\partial \omega / \partial t = \nu \nabla ^2 \omega$, and the heat equation is $\partial T / \partial t = K \nabla ^2 T$. At 1 atmosphere and 400 °K, $K_{water}$ = 23.38 mm$^2$/s and at 300 °K, $K_{air}$ = 19 mm$^2$/s. For PTFE, *K* = 0.124 mm$^2$/s, lower by 1/200th. The heat diffusion distance in cob $\phi$ time period 0.31 s (“Methods”) $\approx$ $O(\sqrt{KT})$ = 2.7 mm, roughly the size of the kernel thickness, and the diffusion time $\approx O(\sqrt{L^2/K})$. When the heat reaches the kernel thickness vertically, one lateral oscillation takes place.

Consider the plumes in the side boundaries in Fig. 7 where there is no solid wall, and yet, vorticity is produced (see the mushroom-shaped distributions in Figs. 6, 7). The spatiotemporal vorticity form of the compressible Navier-Stokes equation and the temporal equation of the vibration of a vertical mechanical system, such as a spring, mass and damper system, are similar^24^. For vanishing inertia forces and spatial variations which apply to the stable time invariant mushrooms of the confined plume^20^, formed impulsively, for a brief time, the compressible vorticity equation reduces to:

1 $$\begin{aligned} \partial \omega / \partial t = (1/(\rho ^2)) \nabla \rho \times \nabla p \end{aligned}$$

where $\nu$ is absent. The damping and the spring constant terms are absent and only the mass term (the buoyancy term) remains; and the external vertical force is absent. This equation shows the temporal generation of vorticity in the confined plume due to the cross product of pressure (*p*) and density ($\rho$) gradients although no wall is present (Figs. 6d, 7). Vorticity is generated due to the misalignment between density and pressure gradients whereby the fluid experiences torque. Also, since the axial pressure and cross sectional area drop, the vortex will spiral, narrow, and elongate allowing wave propagation—displaying elasticity—lower right 2 in Fig. 7b^24,28^. The spiral in Fig. 6a could be due to vertical change in the plume cross sectional area.

Since the acceleration of the fluid parcel in the mushroom-shaped distributions (Figs. 6, 7) is away from the initial position, overturning has occurred, meaning there is convection and $N^2 < 0$, where *N* is the Brunt-Väisälä (BV) angular frequency, imaginary in this case. The air mass is unstable. While BV give the atmospheric or oceanic stratification criterion for instability, the present work regarding thermal stratification, shows how vorticity is produced and maximally stretched with maximum turbulence amplification away from any solid boundary. Such vorticity and turbulence maximization is not along the axis of the plume (in the middle of the frame), but is oscillatory and is along at ± 45° *slants, upward and downward.* Uncommonly, vorticity has been produced away from a solid surface whose intensification is the dominant source of vorticity in the plume.

Using the laminar plume terminologies^20^, Fig. 7 shows a quasi-periodic axial array of vertically dispersed plumes in the middle, and several larger diameter confined plumes laying slanted at ± 45° to the vertical at the cold-warm boundaries on the left and right (Fig. 7c). At the stagnation point, the bifurcation of incoming cold air from the left side replaces the rising warm air, oscillates and then the slanted plumes convect both upward and downward at ± 45°, indicating the critical nature, *Ri* = 1.0 (defined below). On the other hand, in the middle, *Ri* > 1.0. In a three-dimensional shear flow, due to stretching, the vorticity and turbulence amplification reaches a maximum where the vorticity is inclined at 45° (the direction of principal strain)^21–24^.

The dispersed plumes in the cluster in Fig. 7 are narrower and taller than in isolated starting plumes^20^ because the periodic oscillation of the cob organizes the plume cluster and the growth rate of the Kelvin-Helmholtz instability, delaying the formation of successive roll-ups. This type of control is explained theoretically by a self-referential phase reset mechanism as is the case in the aggregation of motor neurons^5,29,30^.

To estimate the cob bulk temperature required to produce the plumes from the Richardson (*Ri*) and Reynolds (*Re*) numbers, define *Ri* as the ratio of the buoyancy to inertia forces $Ri = g(\Delta \rho / \rho _o) d/ \nu ^2$ and *Re* as the ratio of the inertia to viscous forces $Re = vd/\nu$. Take $Ri =1$ and *Re* = 3^20^. Taking *d* = 0.00635 m, for 25 °C ambient, kinematic viscosity $\nu = 1.8444\times 10^{-5}$ m$^2$/s, heat diffusivity $K = 2.1789\times 10^{-5}$ m$^2$/s, density $\rho = 1.1845$ kg/m$^3$, velocity *v* = 0.0029 m/s, we get $\Delta \rho /\rho _o$ = 0.000135; $\Delta \rho$ = 0.001599 kg/m$^3$; plume $\rho _o$ = 1.182901 kg/m$^3$. A bulk temperature difference of 0.5 °C with respect to the ambient temperature can create the transitional plumes. The oscillatory mechanism of heating and evaporation cooling of the leaking kernels by the drawn-in cold air prevents the build up of heat.

## Discussion

(1) We can define control as a state when the goal is to achieve the ratios of any two orthogonal elements of the angular momentum: $I_\phi (t)$, $I_\theta (t)$, $I_\psi (t)$ to remain constant with *t* by devising the inter-component transfers between the elements to $\rightarrow$ 0. (2) The rate of separation of the neighboring state trajectories given by the Lyapunov exponents $\lambda _\theta$, $\lambda _\phi$ and $\lambda _\psi$ $\simeq$ 0, where the trajectory separation is the least in the oblong grape (Fig. 2A). (3) The heat-driven instabilities of plume, corn fibre and lubrication occur simultaneously. (4) Viscous wall-friction is a component of drag in swimming objects. The viscous drag reduction technique of heating, by raising the surface temperature of the swimming object $\rightarrow$ 100 °C, appears feasible in turbulent water flows (heating is known to delay transition) by utilizing the reduction of absolute viscosity of water with rising temperature. In air, instead, surface chilling is required. (5) The work suggests that turbulent boundary layers may be multistable. This proposition would resolve the past controversy of whether inertial or viscous mechanisms are solely responsible for turbulence production in wall-bounded flows, by stating that a turbulent boundary-layer is multistable and not monostable. (6) Further work is needed on the elasticity of small and hot water rich small fruit. (7) The mean kernel popping impulse occurs at 85 ± 1.5 dBA, defined coincidentally as the safe human noise threshold of a young male. The cob oscillation can shift for pops of < 85 ± 1.5 dBA, but is more likely to shift to a new mode for > 85 ± 1.5 dBA. The noisiest pop was at 101.4 dBA, above conversational noise (72 dBA) and background noise (42 dBA) measured in the room. Pumpkin seeds were found to swell and pop (93.6 dBA) with their husks barely opening, also suggesting that popping is a temperature and not pressure dominant mechanism. Controlled heating of submicron bubbles would be useful for insight into the popping mechanism. (8) Commonly, $St_{fA} = fA/U$, where *f*, *A* and *U* are fish parameters^26^. On the other hand, based on the jet half angle $\beta /2$—a small value, define $St_{jet} = 2tan(\beta /2)$^25^. For fish-jet lock-in producing return to initial condition in each cycle in each state variable, when efficiency is maximized, $St_{fA} = St_{jet}$ because the fish tail oscillations are autonomous. Notably, the cobs’ $\theta$ and $\phi$ oscillations in Figs. 3B and 6c, respectively, are also autonomous. In Fig. 3B and inset B, *U*(*x*, *t*) is the cob velocity towards the stem, *A* is the pitch oscillation amplitude and *f* is the frequency of pitch oscillation. This cob pitching oscillation is analogous to fish swimming where the tail flaps and a forward motion is generated due to the symmetry breaking to a reverse Kármán jet^16^. In Fig. 6c, the plume jet angle of the cob $\phi$ oscillation is another independent example that is analogous to a penguin “flying” underwater due to wing flapping, as also with insects. All seven types of fruit display autonomous $\phi$ or $\theta$ oscillations (Figs. 2, 4), and we can expect each to be analogous to fish and penguin swimming. Alternatively, comparing Fig. 3B with C (or Fig. 6c), the $\theta$-based $St_{cob}$ = $\phi$-based $St_{plume}$, for conserved momentum and small $\phi /2$ (5°-10°) assumption; here, avg = average value. Autonomous thrust oscillations are difficult to design in a laboratory, however we have discovered that the seven types of fruit offer a new test bench. (9) Effects of small guided surface irregularities on $R_{\psi drift}$ should be explored.

## Methods

The multistability observations reported are from cobs of corn procured during August-October 2020 when the 90 day average for the Newport County, Rhode Island precipitation/departure were 0.1036 m/– 0.1773 m—near drought conditions. In the present work, the husked cobs are placed on a smooth, level glasstop hotplate. The 6.70 kW, 240 V, 28 A, single phase 60 Hz AC hotplate has a diameter of 225 mm, with an inner circle of diameter 145 mm. The maximum temperature measured with an FLIR infrared camera is 149 °C. The kernel layout is more cambered than the cob. In water, temperature rising to 100 °C, $\mu$ drops to 0.282 Ns/m$^2$, at a rate of 0.003 Ns/m$^2$ per °C = 0.1% per °C. This rate of variation is significant and will affect *h* by the same amount (0.1% per °C) linearly, for *F*, *R* and *U* remaining unchanged.

The aspect ratio (length/maximum diameter) is 1.0 in the blueberries and tropical berries, 1.5 in the grape tomato and red grape, 2.42 in oblong grapes, 3.5 to 4.5 in cobs and 6 to 10 in green chillies. Video 3 compares grape tomatoes with red grapes of the same size and mass (9–10 g, and maximum diameter $\times$ length of 2 cm x 3 cm). In oblong grapes of an average mass of 4.4 g, 37.2 mm length and end diameters of 25.2 mm and 11 mm, Video 3A shows the autonomous yawed rotation at 0.13 m/s with rolling motion. Video 3B shows the autonomous motion in the green chillies of mass 1.5 g and length 5 to 6 cm.

The cob length and circumference are weakly dependent on mass (90–210 g, Fig. 1). Estimated infrequent green kernel popping temperature and pressure are 138 °C and 5 Bar in the present work, while it is 180 °C and 10 Bar in the popping of almost all hard popcorn in the published literature^14^. Yet, the mean sound pressure level (SPL) of pops is similar^18^. The uncertainty in the measurements of SPL is ±1.5 dB. A listing of 11 edited videos appears in the SI.

### Kernel hoop stress scaling

The concentric core and outer cross sections of the cob of annular gap *t*, which is equal to the radial kernel thickness, is filled with steam when hot. Hoop stress is given by $\sigma _H = Pd /2t$, where *P* is the internal pressure, *d* is the core diameter and $t = d$ at all axial cross sections. Take $\sigma _H$ as the yield stress. Assume that a sub-micron size bubble exists at the root of the kernel (a weak point) whose temperature has risen to 149 °C and the critical pressure $P_{cr}$ has risen to 5 Bar = 0.5 MPa. Surface tension will balance the pressure initially. Then, if $P/\sigma _H \ge 2.0$, the bubble will burst making the popping sound (dBA), shown in Fig. 1. The sound wave will reverberate in the kernel chamber, aided by the oscillation of the ejecting hot wet fibre (Fig. SI-1, Video 6). Notably, $\sigma _H$ is independent of the cob dimensions, making the kernels independent, which hints at the similarity of the mean dBA in popcorn. If $\sigma _y$ is yield stress, the kernel pops when $\sigma _H /\sigma _y \ge 1.0$, or $P_{cr} / (2 \sigma _y) \ge 1.0$; then $\sigma _y = 0.25$ MPa. This yield stress is 1/100th of the ultimate strength of rubber. A regular popcorn hull has an ultimate strength $\sigma _c$ of 10 MPa which is similar to that of rubber 10–25 MPa^13^.

### Natural oscillation and friction

Natural oscillation gives the time periods for a linear pendulum with no friction. Neglecting friction, the orthogonal independent $\phi$, $\theta$ time periods of cob oscillation are $T_{ro}$ and $T_{Ro}$ given by the simple harmonic (linear) pendulum such as $T_{Ro} = 2\pi R/g$ and $T_{ro} = 2\pi r/g$. From fits, axial radius of curvature $R = 10r$, where *r* is the cob radius where rolling. Therefore, $T_{Ro}$ = 10$T_{ro}$ and $f_{ro}$ = 10$f_{Ro}$. Note that the $\phi$, $\theta$ frequencies of the wing flapping of swimming and flying animals are the same, however not in the cob. Take *R* = 0.25 m, *r* = 0.025 m. The baseline values would be $T_R$ = 1 s, $T_r$ = 0.31 s; $f_R$ = 1 Hz, $f_r$ = 3.15 Hz. Conversely, since the videos do show oscillation in these ranges, friction is small, and quality factor *Q*—a measure of friction—is high. High *Q* signifies a sharp tuning of the oscillator meaning a narrow bandwidth and a good disturbance rejection. The friction is low because the absolute viscosity of the boundary lubricating water is very low at 100 °C (only 32 times of air).

### Spring constant of cobs

Neglecting damping, the resonant or natural frequency $\omega$ of an oscillator is given by $\omega _o^2 =k/m$, where *k* is the spring constant and *m* is the mass. For $\omega _o =2\pi f_o$, $f_o$ = 1 Hz and *m* = 0.20 kg, *k* = 7.9 kg/s$^2$ = 7.9 N/m = 0.0079 N/mm; and for $\omega _o =2\pi f_o$, $f_o$ = 3.15 Hz and *m* = 0.20 kg, *k* = 12.4 N/m = 0.0124 N/mm—approximately close values.

### Heat modeling

The green kernels mostly leak and do not pop. Kernel water heat content is $Q_{lo} = ms\Delta T$ = (0.17/1000) kg $\times$ 4182 J/Kg/°C $\times$ (((104° to 112°) − 20) °C) = 59.72 J/kernel = *P* (W) × 35 s (Fig. SI-2); power *P* (W) = 1.7 W. Kernel diameter *d* = 0.005 m, on the surface, the low end temperature value $q_{lo}$ = (1.7 W)/($\pi$ (0.005)$^2$ m$^2$) = 1.7/0.0000785 W/m$^2$ = 21,656 W/m$^2$ = 2.16 × 10$^4$ W/m$^2$. Similarly, $Q_{hi}$ = 0.17/1000 kg) × 4182 × (112° − 20°) = 65.4 J; $P_{hi}$ = 65.4/35 = 1.87 W; the high end temperature value $q_{hi}$ = 1.87/0.0000785 = 23,805 = 2.4 × 10$^4$ W/m$^2$. Heat flux *q* is in excellent agreement with the graph of boiling water in Fig. 1^31^.

### Corn grilling efficiency

We calculate the heat retention by corn kernels. Total maximum hotplate power = 6.7 kW. Hotplate area = 3.14 × 0.225 × 0.225/4 m$^2$ = 0.03974 m$^2$. One cob projected area = 0.75$\times LW$ + 0.5W$\times$0.25*L* = 0.75$\times$0.175$\times$(0.175/3.14) + 0.5$\times$(0.175/3.14)$\times$0.175 × 0.25 = 0.007315 + 0.001219 = 0.008534 m$^2$. Ring to cob area ratio = 0.03974/0.008534 = 4.66. Number of kernels = 120. Watts to kernels = 120 × 1.87 = 224.4 W. Kernel heat power = 1.87 W/kernel. For 4.66 such cobs, Watts = 4.66 × 224.4 = 1.05 kW = 16% of the total electrical power in. The cob grilling efficiency, or heat retention, is 16%.

### Heat-surface tension oscillation in green kernel bubble

In the kernels not popping but making an outgrowth of a kernel size bubble (Fig. SI-2), the latter can be modeled as follows. An instability scaling relationship can be written by equating heat energy on the surface to the surface tension effect, $qd^2 = Tgtd$, left term = W/m$^2$ × m$^2$ = W; right term = N/m × m/s$^2$ × s × m = Nm/s = W. Pressure inside the bubble in Fig. SI-2 is *T*/*d* N/m$^2$ = 0.728/0.005 = 145.6 N/m$^2$ = 145.6/101325 = 0.001437 atmosphere, a small increase above atmospheric pressure.

## Supplementary Information

Supplementary Information 1.
