# Supplementary material for: Multistable autonomous motion of fruit on a smooth hotplate
Source: Sci Rep. 2022 Jan 7;12:20. doi: 10.1038/s41598-021-03859-8 (PMC8742060; doi:10.1038/s41598-021-03859-8)
Supplement: Supplementary file 1 — Supplementary Information 1. [file 41598_2021_3859_MOESM1_ESM.pdf]

SUPPLEMENTARY INFORMATION (SI):
Multistability

Promode R Bandyopadhyay
153 Chases Lane, Middletown, RI 02842

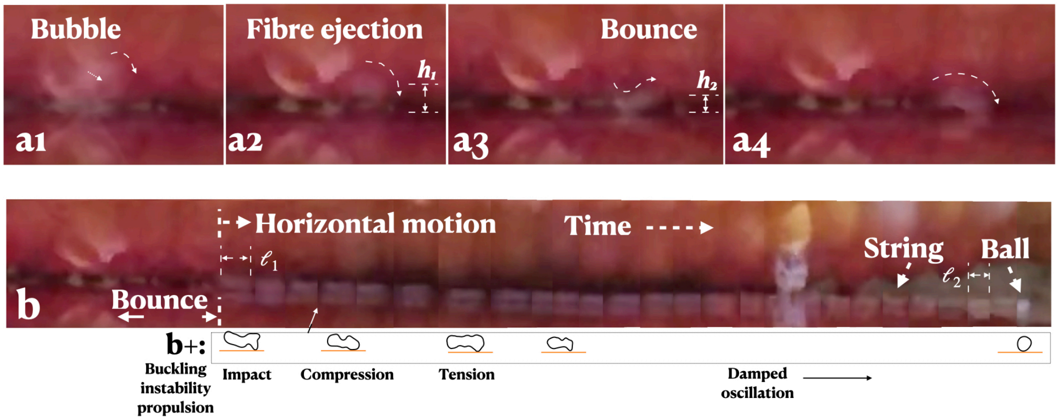

Fig. SI-1. Elasticity of corn fibre.

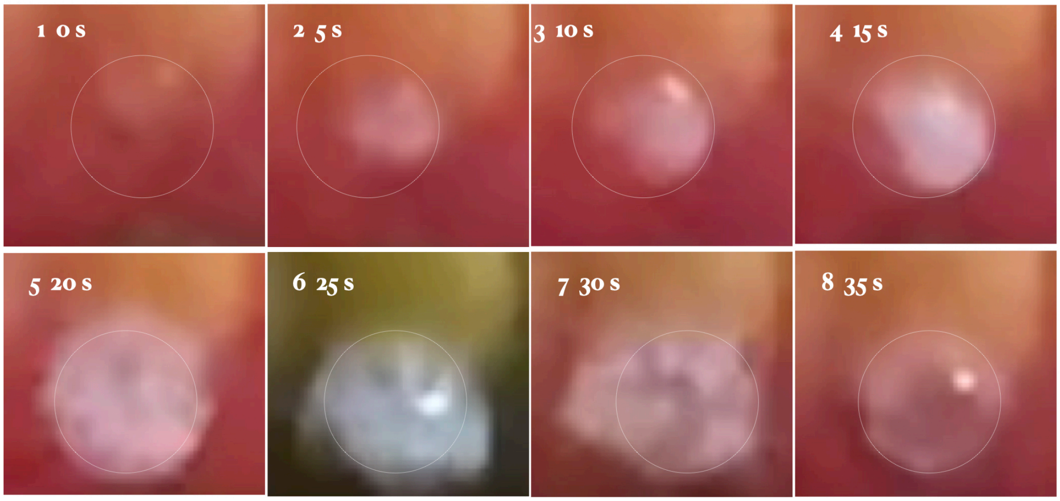

Fig. SI-2. Effect of heat on kernel water showing nucleation and coalescence. See boiling graph figure 1 in article for onset of bubble formation. The dotted circle is added to show the departure from surface tension induced equilibrium. x4.

Table. 1. List of Submitted Videos.

Web link for access: https://drive.google.com/drive/folders/167c7L3enIj22gW805pDbNn4n4VkzywOb?usp=sharing

Video #	File name	Format	Size	Description
Video 0	VIDEO 0 COMPOUND PENDULUM	mp4 MPEG-4 MOVIE	326.5 MB	Reference nonlinear oscillator showing initial condition dependence; unrepeating trajecories
Video 1	VIDEO 1 CORN ROLLING	mp4 MPEG-4 MOVIE	12.7 MB	CORN ON COB: Elementary rolling
Video 2	VIDEO 2 CORN PITCHING	mp4 MPEG-4 MOVIE	12.3 MB	CORN ON COB: Elementary pitching
Video 3	VIDEO 3 ROLLING GRAPE AND GRAPE TOMATO	mp4 MPEG-4 MOVIE	41.7 MB	GRAPE TOMATO and RED GRAPE: elementary rolling and yawing
Video 3A	VIDEO 3A OBLONG GRAPE AUTONOMOUS ROLLING AROUND THE GAP BETWEEN THE OUTER AND INNER HEATING RINGS			MOONDROP OBLONG GRAPES
Video 3B	VIDEO 3B GREEN CHILLIE'S AUTONOMOUS ROLLING AND YAWING			GREEN CHILLI
Video 4	VIDEO 4 TROPICAL BERRY AND BLUEBERRY Firm versus Soft Multistability	mp4 MPEG-4 MOVIE	35.6 MB	BERRIES-Blue (tender) and Tropical (firm): Multistability
Video 5	VIDEO 5 CORN SPINNING IMG_0852 POSITIVE FEEDBACK LOOP LowRez	mp4 MPEG-4 MOVIE	84.6 MB	CORN ON COB: Yawed rotation and axial crawling
Video 6	VIDEO 6 ELASTICITY IMG_0860 ejection ball bounce 10slow motionFlashFreezerame copy	.mov	2.02 GB	CORN ON COB ELASTICITY: Bounce
Video 7	VIDEO 7 STABILITY SHIFTING GRADUAL IMG0779LOWREZEditeddBACrlnShiftingfromRollingToPitching copy	mp4 MPEG-4 MOVIE	104 MB	CORN ON COB: Gradual shift in stability
Video 8	VIDEO 8 IMG_0851PopCorelationRotation copy	.mov	10.93 GB	CORN ON COB: Correlation of motion shift with pop dBA
